# Intratumor microbiota as a novel potential prognostic indicator in mesothelioma

**DOI:** 10.3389/fimmu.2023.1129513

**Published:** 2023-03-14

**Authors:** Francesca Pentimalli, Marija Krstic-Demonacos, Caterina Costa, Luciano Mutti, Emyr Yosef Bakker

**Affiliations:** ^1^ Department of Medicine and Surgery, LUM University “Giuseppe DeGennaro”, Bari, Italy; ^2^ Biomedical Research Centre, School of Science, Engineering and Environment, University of Salford, Salford, United Kingdom; ^3^ Cell Biology and Biotherapy Unit, Istituto Nazionale Tumori-Scientific Institute for Research and Care (IRCCS)-Fondazione G. Pascale, Napoli, Italy; ^4^ Center for Biotechnology, Sbarro Institute for Cancer Research and Molecular Medicine, College of Science and Technology, Temple University, Philadelphia, PA, United States; ^5^ Department of Biotechnological and Applied Clinical Sciences, University of Aquila, L'Aquila, Italy; ^6^ School of Medicine, University of Central Lancashire, Preston, United Kingdom

**Keywords:** mesothelioma, microbiota, microbiome, bioinformatics, Kaplan-Meier, DEG (differentially expressed gene) analysis, functional annotation analysis, Cox proportional hazards modelling

## Abstract

**Introduction:**

Despite increased attention on immunotherapy, primarily immune checkpoint blockade, as a therapeutic approach for mesothelioma (MMe), its efficacy and tolerability remain questioned. One potential explanation for different responses to immunotherapy is the gut and intratumor microbiota; however, these remain an underexplored facet of MMe. This article highlights the cancer intratumor microbiota as a novel potential prognostic indicator in MMe.

**Methods:**

TCGA data on 86 MMe patients from cBioPortal underwent bespoke analysis. Median overall survival was used to divide patients into “Low Survivors” and “High Survivors”. Comparison of these groups generated Kaplan-Meier survival analysis, differentially expressed genes (DEGs), and identification of differentially abundant microbiome signatures. Decontamination analysis refined the list of signatures, which were validated as an independent prognostic indicator through multiple linear regression modelling and Cox proportional hazards modelling. Finally, functional annotation analysis on the list of DEGs was performed to link the data together.

**Results:**

107 genera signatures were significantly associated with patient survival (positively or negatively), whilst clinical characteristic comparison between the two groups demonstrated that epithelioid histology was more common in “High Survivors” versus biphasic in “Low Survivors”. Of the 107 genera, 27 had published articles related to cancer, whilst only one (Klebsiella) had MMe-related published articles. Functional annotation analysis of the DEGs between the two groups highlighted fatty acid metabolism as the most enriched term in “High Survivors”, whilst for “Low Survivors” the enriched terms primarily related to cell cycle/division. Linking these ideas and findings together is that the microbiome influences, and is influenced by, lipid metabolism. Finally, to validate the independent prognostic value of the microbiome, multiple linear regression modelling as well as Cox proportional hazards modelling were employed, with both approaches demonstrating that the microbiome was a better prognostic indicator than patient age or stage of the cancer.

**Discussion:**

The findings presented herein, alongside the very limited literature from scoping searches to validate the genera, highlight the microbiome and microbiota as a potentially rich source of fundamental analysis and prognostic value. Further in vitro studies are needed to elucidate the molecular mechanisms and functional links that may lead to altered survival.

## Introduction

MMe is a rare cancer that may arise in the pleura, peritoneum, pericardium, or tunica vaginalis, with most cases affecting the pleura (1). MMe has historically been characterized by an exceptionally poor prognosis with limited treatment options that largely consisted of first-line anti-folates in combination with platinum-based therapy. Immunotherapy, particularly immune checkpoint blockade, has been investigated in the context of MMe. Although first-line combination of the immune checkpoint inhibitors (ICIs) nivolumab (anti-PD-1) and ipilimumab (anti-CTLA-4), based on the CheckMate 743 trial (2) has been approved for MMe, its efficacy has been questioned, with two comparative studies that have shown no survival benefit in the CheckMate 743 trial relative to trials studying cisplatin + pemetrexed + bevacizumab against cisplatin + pemetrexed (3, 4). One of these studies also casts a doubt on the combination of durvalumab and chemotherapy (4). Moreover ICIs have shown no significant superiority on standard treatment, either from real-world analysis (5) or in second-line settings (6). Thus, there is a need to investigate immunotherapy at a molecular level in mesothelioma, to further elucidate potential mechanisms and improve outcomes (7).

One potential reason for the varying efficacies of immune checkpoint blockade is the gut microbiome (8–10). Microbiome and microbiota are often used interchangeably, but the difference between the terms is that microbiome refers to “the collective genomes of microorganisms in a particular environment”, whilst microbiota refers to “the community of microorganisms themselves” (11).

The microbiota consists of a vast collection of commensal archaea, bacteria, fungi and viruses that shows significant intrapopulation variation (9). When the microbiota is in balance with the host, a condition of eubiosis, it contributes to body homeostasis and to a healthy immune system, whereas microbial dysbiosis—the imbalance of microbiota with harmful species outcompeting benign (12)—contributes to the pathogenesis of many diseases including cancer. Indeed, beyond the well-recognized role of the gut microbiota in health and disease, in the past decade many studies have demonstrated the presence of a live and active intratumor microbiota which can affect disease progression and the therapeutic response (13, 14). Despite the rising recognition of the importance of gut and intratumor microbiota in cancer, their presence and impact in MMe remain significantly understudied. As of 15^th^ December 2022, there were only ten peer-reviewed publications in PubMed for the search terms “((microbiota OR microbiome) AND mesothelioma)”. Of these, only four actually contained clear and pertinent information on MMe and the microbiota/microbiome (15–18), with the rest as text-mining artefacts.

It is noteworthy that none of these studies have explored a link between the microbiota/microbiome and clinical characteristics in patients with MMe. Therefore, given the very limited literature related to the microbiome in MMe and the potential role it may play in the response to ICIs, there is evidently a need to investigate this further. To address this, herein TCGA intratumor microbiome data from MMe patients has been investigated in association with patients’ clinical characteristics. We find that, upon dividing the patients into “Low Survivors” and “High Survivors”, the only clinical characteristic that significantly differs between them was histological subtype, with epithelioid being more common in “High Survivors” versus biphasic in “Low Survivors”. Additionally, we identify 107 genera signatures that are significantly associated with survival, with only 27 genera returning published papers following a scoping search for each genus and cancer, and only 1 genus (*Klebsiella*) returning a published result for mesothelioma. Tying the intratumor microbiome data with the cancer cell data is that fatty acid metabolism was the most enriched functional annotation in the “High Survivors” group (based on differential gene expression analysis between the two groups), a process that is known to have two-way interplay with the microbiome.

## Methods

### Overall workflow

**Figure 1:**
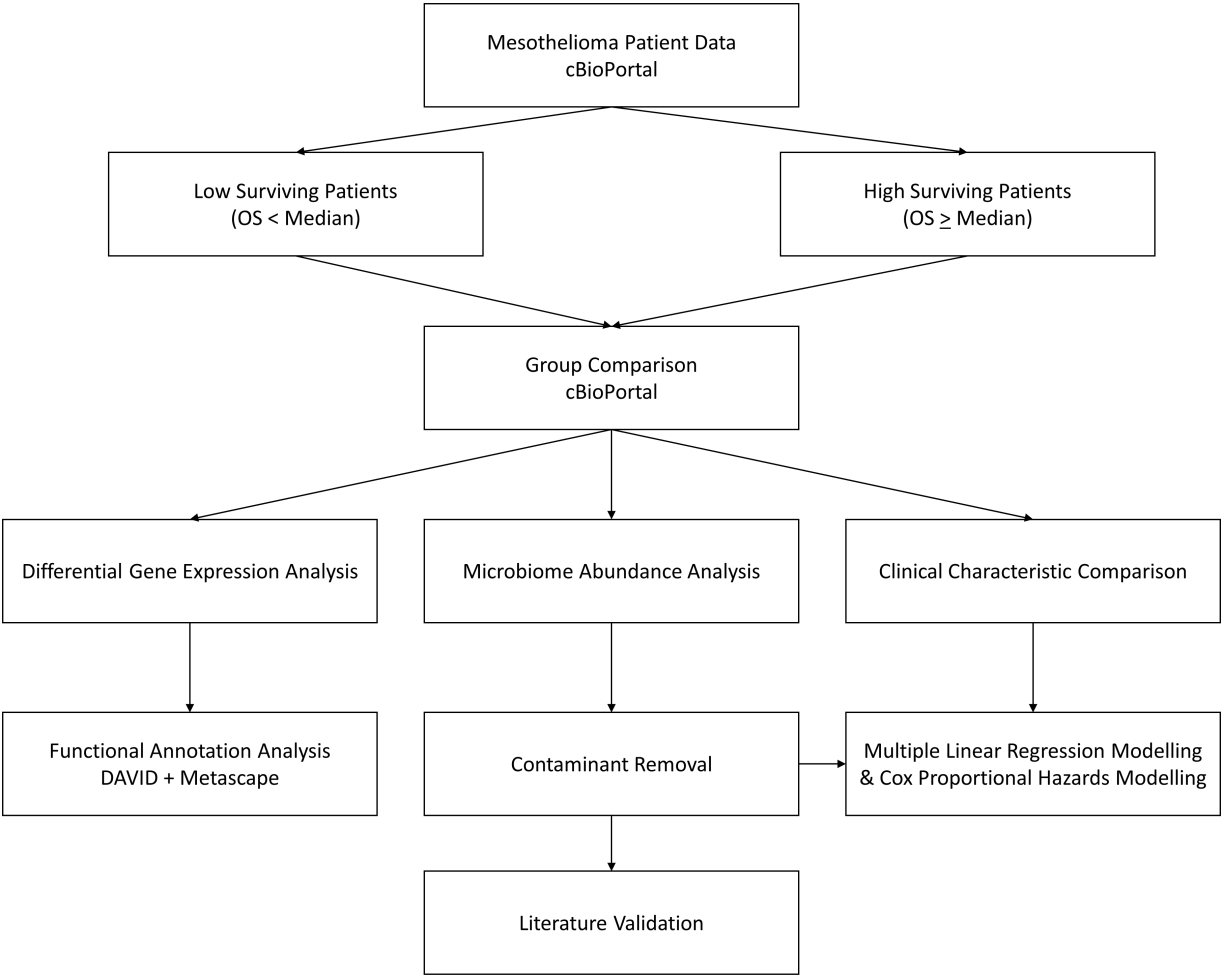
Workflow diagram. cBioPortal represents the starting point, where MMe patient data is accessed and patients are divided by the median overall survival. These two groups are then compared to assess differences in clinical characteristics, identify differentially expressed genes, and identify differential abundance in microbiome signatures, which is taken forward for contaminant removal, literature validation, and survival modelling.

Further detail is provided in subsequent headings, but the overall workflow for this study can be seen in **Figure 1**.

### Study selection and patient grouping

The cBioPortal database (19, 20) was utilized to interrogate MMe patient data (date of access 05 April 2022). The “Mesothelioma (TCGA, PanCancer Atlas)” study was selected because it included the highest number of patients together with the pertinent intratumor microbiome signatures and survival data required for the study. The other study with the same number of patients (“Mesothelioma (TCGA, Firehose Legacy)”) lacked usable survival data (21), whilst the third study (“Pleural Mesothelioma (NYU, Cancer Res 2015)”) had only 22 patients and did not have microbiota/microbiome data available (19, 20, 22).

After selecting the “Mesothelioma (TCGA, PanCancer Atlas)” study and choosing “Explore Selected Studies”, patient IDs and survival lengths were downloaded to be analysed outside of the cBioPortal platform. After discarding the individual patient whose OS_MONTHS (overall survival in months) value was “N/A”, the median OS_MONTHS value was calculated from the remaining patients (n=86). Patients were then divided into “Low Survivors” (OS_MONTHS less than the median) or “High Survivors” (OS_MONTHS greater than or equal to the median).

### Identifying microbiome differences

After identifying the patient subgroups described above, the cBioPortal database was accessed once more with the “Mesothelioma (TCGA, PanCancer Atlas)” study. The subgroups were regenerated on the cBioPortal platform *via* the “Custom Selection” (based on Patient ID) and “Groups” functions. After regenerating the subgroups, they were analysed using the “Compare” cBioPortal function under the Groups setting.

This analysis automatically generated the Kaplan-Meier survival curve between the two groups, alongside the microbiome signatures comparison. In order to calculate a more precise p-value alongside the hazard ratio for the survival data, the resultant raw Kaplan-Meier data was downloaded and input to KMPlot using the upload function (23, 24). The microbiome signatures data were originally added to cBioPortal for a number of cancers on the basis of another study, which has since been retracted based on criticisms from Gihawi and colleagues (https://pubmed.ncbi.nlm.nih.gov/37811944/). Although the authors’ rebuttal in which they said they reproduced their findings remains live (https://www.nature.com/articles/s41388-024-02974-w), it is worth noting that this retraction means that some specific information in this manuscript (e.g. exact genera) may be uncertain, even if there may still be merit in the overarching findings.

Clinical parameters for patients were also obtained via the Compare analysis, as were the differentially expressed genes.

Whilst exploratory studies such as the analysis contained herein are not strictly required to perform multiple comparison corrections (26, 27), microbiome signatures were only taken further if they were significant based on q-value (q<0.05). This permitted a greater focus on those genera that were more likely to have links to patient survival. The same was true for the identification of differentially expressed genes.

### Functional annotation analysis

In order to interrogate the differentially expressed genes identified above and how they may relate to survival, the DAVID (28, 29) and Metascape (30) tools were employed. Gene lists that were highly expressed in both the low surviving and high surviving patient groups were in turn entered into each tool to identify clusters of functional annotations and enriched annotations.

### Contaminant removal

Due to the recognised issue of contaminants (i.e., tumour sample contamination by external microbes during data collection and processing) when considering microbiome data, a decontamination analysis was performed on the list of genera that were statistically significantly associated (based on q-value) with patient survival.

This was performed through cross-examination with supplementary information from the aforementioned original article which added the signatures to cBioPortal.

### Literature scoping of genera

The final list of genera identified in the previous step were collated into a table after which searches were conducted to assess the breadth of literature pertaining to each genus. Searches were performed on PubMed (date of access 25^th^ April 2022 – 7^th^ July 2022) using the Boolean operator AND in the below format:

[Genus Name] AND Mesothelioma

[Genus Name] AND cancer

For the genera that had “Candidatus” in their name, searches were performed with and without the “Candidatus_” prefix to ensure searches were as exhaustive as possible. The literature scoping allowed for the identification of the breadth of knowledge related to each genus in both MMe and cancer in general.

### Multiple linear regression modelling of putative prognostic factors

To determine the independent prognostic value of the microbiota identified in the previous step, multiple linear regression modelling was employed. To begin, the full microbiome abundance values (per patient, in log RNA Seq CPM) for all 1406 genera was downloaded from cBioPortal, alongside known clinical parameters such as overall survival (months), age, stage, and tumor histology (19, 20). This microbiome data was then filtered to include only the genera identified in the previous step, which were then subdivided into “good genera”—those identified to be more abundant in High Survivors than Low Survivors—and “bad genera”—those identified to be more abundant in Low Survivors than High Survivors.

It is known that inclusion of too many covariates on a multiple regression model can lead to overfitting, where the model on the surface appears to predict the outcome variable well, but in fact is responding only to noise (31–33). To avoid this problem, the log RNA Seq CPM values for all “good genera” were summed to an individual value per patient (“Positive Microbiome Value”), with the same step performed for the “bad genera” (“Negative Microbiome Value”).

Other parameters commonly thought to influence prognosis—namely age, stage, and tumor histology—were also considered. The age values for each patient were taken as-is, whilst the staging information was simplified to include only the numbers (e.g. 1A and 1B under Neoplasm Disease Stage American Joint Committee on Cancer Code both became 1). It should be noted that this simplification applied only to three patients, as the remainder were simply Stage I, Stage II, Stage III, or Stage IV. Tumor histology was converted to a binary dummy variable (34), with 0 being epithelioid histology whilst 1 indicated non-epithelioid histology. The rationale for this division was the clinical reality that epithelioid patients have significantly better outcomes than non-epithelioid patients (35).

The dependent (outcome) variable for the multiple linear regression model was the overall survival of the patients in months. The initial independent variables were age, stage, histology, Positive Microbiome Value and Negative Microbiome Value. The initial multiple linear regression model was then refined through several iterations (e.g. removal of independent variables) by examination of the resultant adjusted R^2^ values, alongside the p-values for the individual independent variables that were produced at each stage. High p-values were removed on subsequent iterations of the multiple linear regression model.

### Cox proportional hazards modelling

To further validate the potential of the microbiome as a prognostic indicator using an independent method, Cox proportional hazard modelling was employed (36). The same data (age, stage, histology, Positive Microbiome Value, and Negative Microbiome Value) was used for this as in the multiple linear regression model above. Overall Survival Status (i.e. 0 (living) and 1 (deceased)) was also extracted from cBioPortal for each patient (19, 20). These data were input to SPSS, with overall survival (in months) used as the “Time” variable and overall survival status used as the “Status” variable. 1 (deceased) was used as the event for Status. As explained above, age, stage, histology, positive microbiome and negative microbiome were all used as covariates.

## Results

### Validation of survival difference

**Figure 2:**
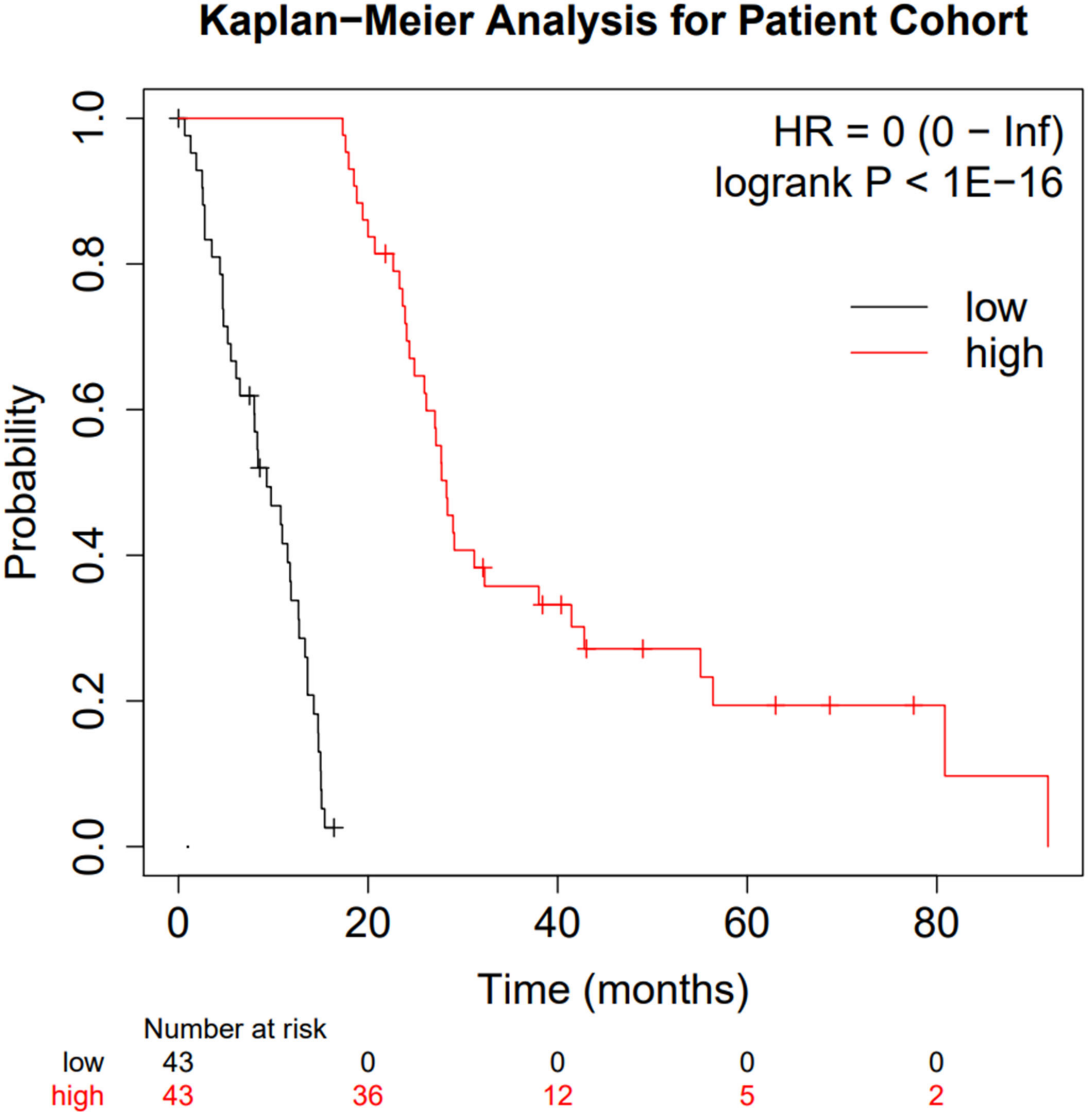
Kaplan-Meier curve comparing the two patient subgroups. Low surviving patients are shown in black whilst high-surviving patients are shown in red.

After generating the “Low Survivors” and “High Survivors” groups described in the Methods above, the survival difference was analysed *via* a Kaplan-Meier curve to validate the grouping approach and ensure the integrity of downstream analysis (**Figure 2**).

**Figure 2** clearly highlights the survival difference between the two groups (p < 10^-16^ and hazard ratio of zero). Whilst clearly an expected result, the significance of the survival difference validates the downstream comparison.

Although the diagnosis age, cancer stage, gender, and histological subtype are of known importance for MMe, there was no statistically significant difference for any of these parameters (based on p- and q-values; see **Supplementary Figures 1**–**7**) except the histological subtype, with biphasic MMe being more common in the “Low Survivors” group as opposed to the “High Survivors” group alongside epithelioid histology being less common in the “Low Survivors” group (**Supplementary Figure 7**). It should be noted that there was the presence of the 9050/3 (Mesothelioma, malignant, NOS) group. This group contains mesothelioma patients who were diagnosed with mesothelioma but with no further information on their histology (NOS = Not Otherwise Specified) (37), but there was not a difference between “Low Survivors” and “High Survivors” for this subtype designation (**Supplementary Figure 7**). Epithelioid histology being less common in “Low Survivors” whilst biphasic was more common is consistent with known literature that epithelioid histology has the best prognosis of the different histological types of mesothelioma (35).

### Microbiome analysis

**Figure 3:**
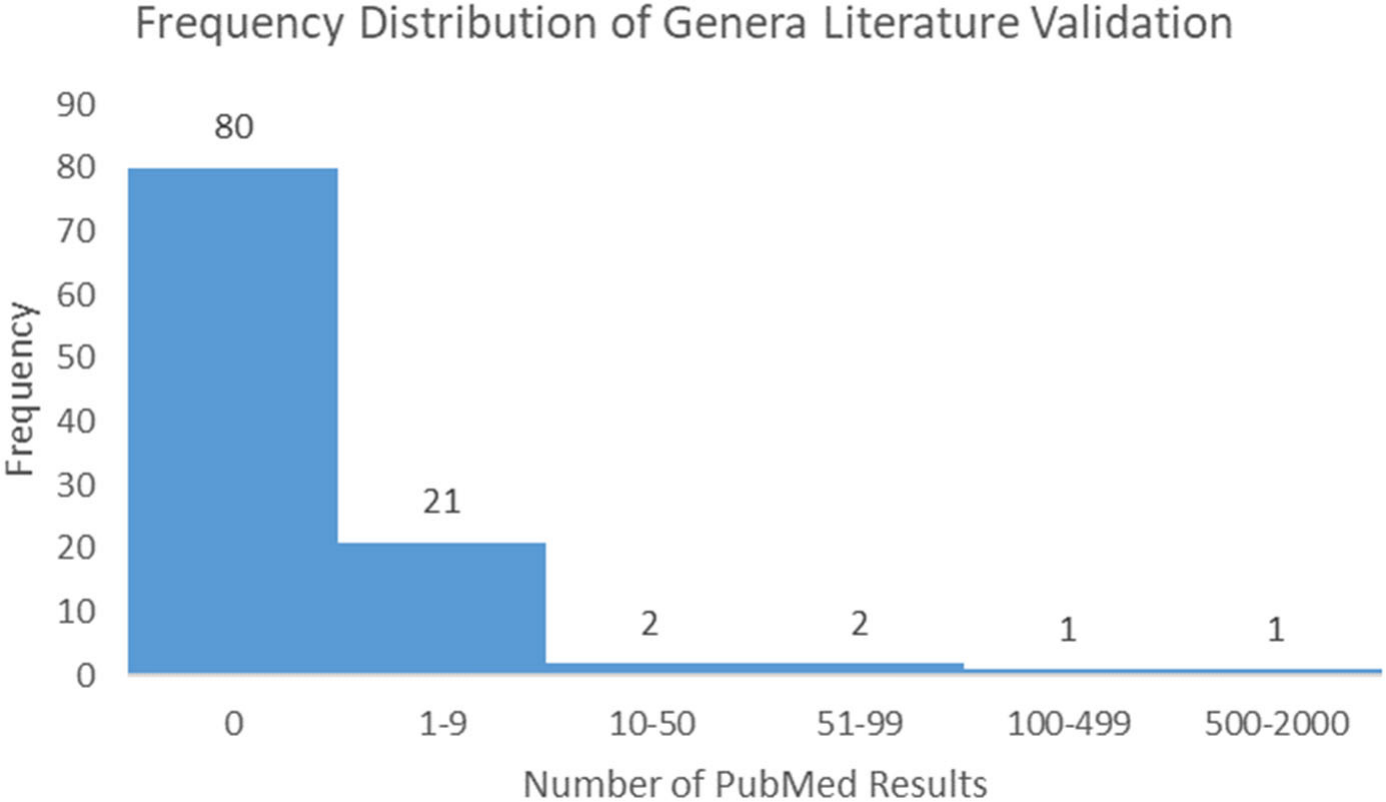
Frequency distribution of PubMed results for genera and cancer.

Following the process described in the Methods, 175 microbiome signatures (genera) were initially identified to be differentially abundant between the “Low Survivors” and “High Survivors” groups (q < 0.05). After decontamination analysis, this number was reduced to 107, of which four genera were more abundant in low survivors and 103 were more abundant in high survivors. Literature scoping highlighted that only one genus (*Klebsiella*) returned an article in association with mesothelioma, whilst even a broader general cancer search still yielded very few results (**Supplementary Table 1**). **Figure 3** below demonstrates the frequency distribution of the number of results returned in PubMed for the genera and cancer in general:

The relative scarcity of literature for the genera and cancer, and especially so for the genera and mesothelioma, highlights the significant infancy of this field, and warrants further investigation. As highlighted, only one genus (*Klebsiella*) returned papers for MMe. Conversely, when searching for cancer in general, 27 genera returned papers. Ranked in order from most to least papers, these were *Klebsiella, Lambdalikevirus, Cyclobacterium, Achromobacter, Yatapoxvirus, Leeia, Magnetococcus, Leptonema, Pragia, Candidatus_Arthromitus, Closterovirus, Vagococcus, Microchaete, Cetobacterium, Chelativorans, Sulfuricurvum, Actinopolymorpha, Cycloclasticus, Beggiatoa, Thalassospira, Pleurocapsa, Anaerofustis, Dichelobacter, Yokenella, Crinivirus, Thioalkalimicrobium*, and *Gemmata*.

### Differential gene expression and functional annotation analysis

**Figure 4:**
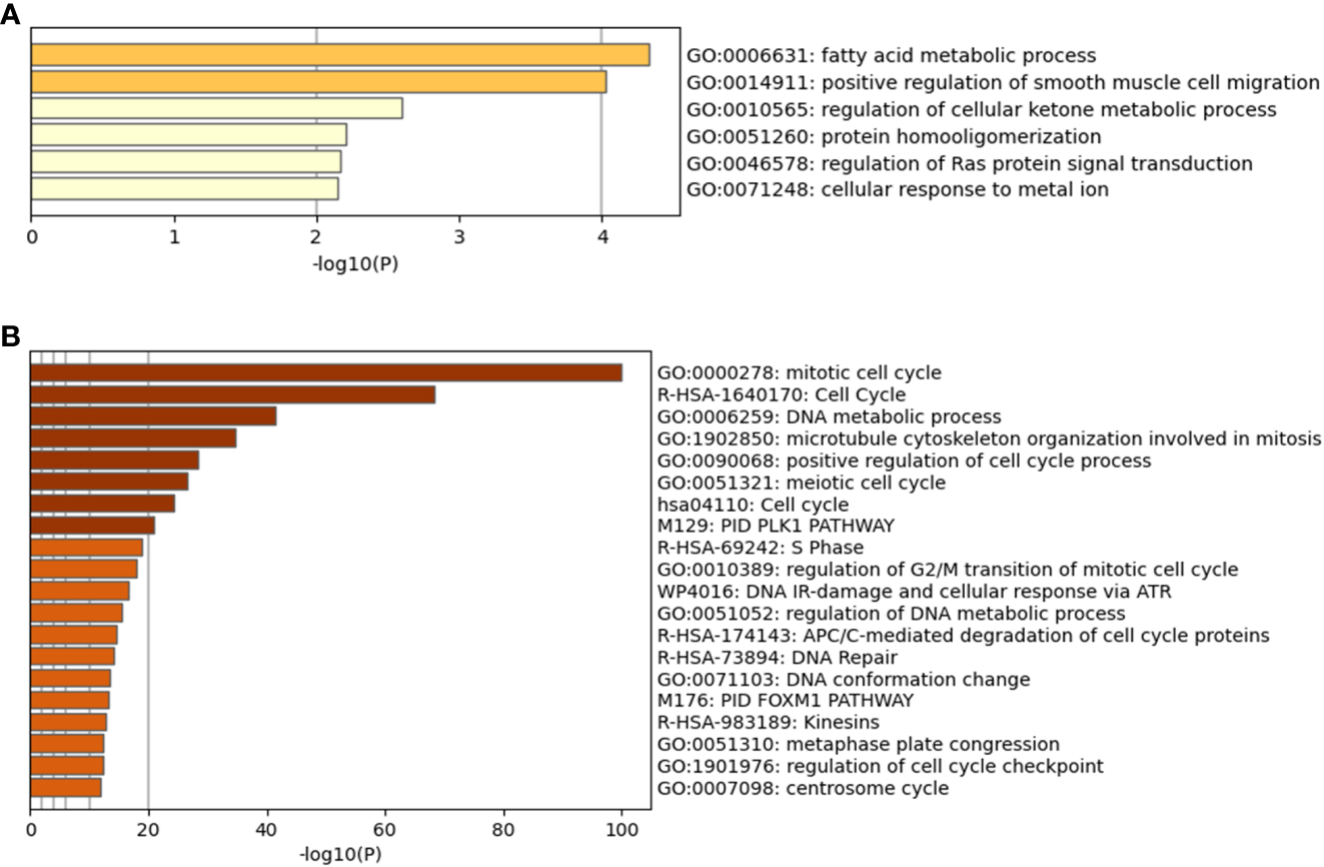
Metascape analysis for differentially expressed genes. **(A)** is the High Survivors group, whilst **(B)** is the Low Survivors group.

Following the approach described in the Methods and based on q<0.05, a total of 60 genes were identified to be significantly more expressed in the “High Survivors” group whilst 274 were significantly more expressed in the “Low Survivors” group, listed in **Supplementary File 1**. To assess the functional relevance of these genes, the DAVID (28, 29) and Metascape (30) tools were accessed, with the latter shown in **Figure 4**.

In addition to the figures generated by Metascape, DAVID analysis identified 64 clusters of annotations for the genes upregulated in the Low Survivors group, with the top three containing terms related to cell division and DNA repair (**Supplementary File 1**). Comparatively, DAVID identified ten clusters of annotations for genes upregulated in the High Survivors group, with the most enriched cluster containing lipid metabolism (**Supplementary File 1**). Thus, the DAVID analysis complements the Metascape analysis, highlighting the distinct biological processes that are overrepresented in each group.

### Multiple linear regression modelling

As described in the Methods, multiple linear regression modelling was performed to identify the independent prognostic value of the microbiome in mesothelioma. The first iteration of the model—”Model 1”—incorporated the patients’ age, stage, tumor histology, Positive Microbiome Value (the sum abundance of the 103 identified to be significantly more abundant in High Survivors), and Negative Microbiome Value (sum abundance of the 4 genera identified to be significantly more abundant in Low Survivors). **Table 1** below summarizes the iterations (refinement) of the model, the independent variables they include, alongside the adjusted R^2^ values and independent variable p-values.

**Table 1:** Multiple linear regression model and iterations.

Model Number	Independent Variables Included	P-Values for Independent Variables (*≤0.05)	Adjusted R^2^ Value for Model	Independent Variables Removed for Subsequent Model & Why
**Model 1**	Age	0.506623975	0.170160087	Negative Microbiome Value – with only four genera adding to its value, it was unlikely to show significant differences
	Stage	0.701255523		
	Histology	0.067083872		
	Positive Microbiome Value	0.003256009*		
	Negative Microbiome Value	0.368887571		
**Model 2**	Age	0.567797635	0.17203895	Age and Stage—as p-values remained high despite previous refinement (in fact, they increased)
	Stage	0.764346949		
	Histology	0.017645789*		
	Positive Microbiome Value	0.000343888*		
**Model 3**	Histology	0.0064759*	0.188463827	N/A
	Positive Microbiome Value	0.000318305*		

Model number, independent variables included alongside their p-values are provided, as well as the adjusted R^2^ value for the model. All models used overall survival (in months) as the dependent (outcome) variable. Raw data behind the models can be seen in **Supplementary File 2**.

As highlighted above in **Table 1**, despite the low adjusted R^2^ indicating that the independent variables explain at most 18.8% of the variation in overall survival, it remains clear that—at least for this patient cohort—the Positive Microbiome Value was the best predictor for overall survival (based on p-value). This was true against known prognostic factors, including age, stage, and tumor histology.

### Cox proportional hazards modelling

To independently validate the prognostic value of the microbiome further using an additional method, Cox proportional hazards modelling (also known as Cox regression) (36) was employed. The same data as for the multiple linear regression model above was used, with the only additional input being the overall survival status (0=alive; 1=deceased) for each patient. The p-value for the Cox proportional hazards model when compared to a null model was <0.001, indicating significant predictive utility. **Table 2** below summarizes the coefficients, p-values, hazard ratios, and 95% confidence intervals for each input variable:

**Table 2:** Cox proportional hazards modelling.

Variables in the Equation	Coefficient (B)	Sig.	Hazard Ratio (Exp(B))	95.0% CI for Exp(B)	
				Lower	Upper
**Positive Microbiome Value**	-0.014	**0.001**	0.986	0.978	0.995
**Negative Microbiome Value**	0.093	0.097	1.097	0.983	1.224
**Age**	0.011	0.467	1.011	0.981	1.042
**Stage**	-0.087	0.534	0.916	0.696	1.207
**Histology**	0.577	**0.045**	1.781	1.013	3.131

Coefficients, p-values, hazard ratios, and 95% confidence intervals for each variable are shown. Statistically significant p-values are in bold.

Consistent with the multiple linear regression model, only Positive Microbiome Value and Histology were significantly associated with survival in this patient cohort. Positive microbiome had a negative coefficient and a hazard ratio significantly below 1 (based on the 95% confidence interval), emphasizing the protective role of these genera. Comparatively, histology (which was a dummy variable with zero for epithelioid and one for non-epithelioid) had a positive coefficient and a hazard ratio significantly above 1 (based on the 95% confidence interval). Thus, it is again demonstrated that non-epithelioid histology is a negative prognostic factor, consistent with previous literature (35).

## Discussion

This study interrogated existing and publicly available patient data with a novel analytical approach to identify genera that were associated with patient survival. It is clear, despite the rising importance of the microbiome in cancer, that the microbiome remains a factor that is highly under-investigated. This is true for cancer in general, with only 27 of the 107 genera identified herein having published literature surrounding them in the context of cancer. The statement of the microbiome being under-investigated is particularly true for MMe, where only one genus out of 107 had literature returned resulting from the search.

*Klebsiella*, whose signature was more abundant in low survivors, returned only three papers in the context of MMe. However, analysis of these papers further highlights the very limited knowledge that exists around the microbiome in MMe. The first study (38) was a case report highlighting incidence of cerebral air embolism in a patient with chronic hydropneumothorax secondary to epithelioid MMe following pleural catheter insertion. Whilst case reports are naturally limited, the only mention of *Klebsiella* was detailed in the pleural fluid culture, where *Klebsiella oxytoca* and *Enterococcus faecalis* were identified. However, it was not stated if this originated from the pleural fluid or if it was a potential contaminant from the catheter. Thus, it is highly probable that these genera in this instance were not associated with the intratumor microbiome.

The second paper returned from the search for *Klebsiella* and MMe highlighted sputum-obtained *Klebsiella pneumoniae* from a MMe patient (39). However, this detection did not describe the link to the cancer, only that it was detected in the patient, and may in fact have originated from an upper respiratory infection. The third and final paper that was returned described a novel compound that had demonstrated efficacy against both microbes (including *Klebsiella*) and MMe cells cultured *in vitro* (40). However, no link was made between *Klebsiella* and MMe.

It is evident from the above that there is currently no literature explaining why the microbiome signature of *Klebsiella* was more abundant in low-surviving patients. The fact that the remaining 106 genera had zero papers returned from the literature search highlights the degree of under-exploration that the microbiome suffers in MMe.

Interrogation of the wider literature around *Klebsiella* in other types of cancers highlights some findings that may be of note. In the case of lung cancer, from the analysis of the microbiome in 67 patients with adenocarcinoma (AD) and 47 cases with squamous cell cancer (SCC), *Klebsiella*, alongside *Acidovorax, Rhodopherax* and *Anerococcus* were identified. These genera were found to be more significantly present in SCC than in AD. In addition, the bacterial flora of patients with lung cancer consists mainly of *Proteobacteria* (especially *Acinetobacter* and *Acidovorax*) with a reduced presence of the genus *Firmicutes* (such as *Streptococcus*) and *Bacteroidetes* (*Prevotella*); instead they were present in the flora of patients with pulmonary emphysema. This composition is different in smoking patients with lung cancer, thus attributing an important role to smoking in carcinogenesis and microbiome change. Of note, smoking patients not only had these more abundant genera, but TP53 mutations in the tissue of these subjects also correlated with impaired epithelial function in the lung and thus with the change in the microbiome (41–43). Furthermore, polyketide synthase positive strains of *E. coli* and *K. pneumoniae* (this locus codes for the bacterial toxin colibactin) were isolated in samples from patients with colorectal cancer. This expression has been related to *K. pneumoniae* hypervirulence and intestinal mucosal invasion (44). Finally, it should be noted that a retrospective study revealed that adjuvant treatment with gemcitabine improves survival in *K. pneumoniae*-negative pancreatic cancer patients, whereas adjuvant treatment with quinolones (which are bactericidal) was associated with better overall survival (OS). This result suggests that the presence of *K. pneumoniae* may promote chemoresistance to adjuvant gemcitabine in pancreatic cancer (45). Taken together, is evident that the wider literature supports the negative impact *Klebsiella* has on patients, which is consistent with our finding that *Klebsiella* was more abundant in Low Survivors than High Survivors.

The independent prognostic value of the microbiome was validated through the multiple linear regression model (**Table 1**). It may initially be surprising that neither age nor stage were validated as predictors of overall survival; however, examination of the underlying data (**Supplementary File 2**) alongside access of the wider literature highlights that this may not be unusual. The 86 patients included within this study were relatively uniform in age; the median age was 64 (mean 63.08) with a standard deviation of 9.78. The more restricted variability in age could help explain the lack of predictive utility for this variable. Similarly, the number of patients at different stages were uneven: ten patients were Stage 1; sixteen patients were Stage 2; forty-four patients were Stage 3; and sixteen patients were Stage 4. This again indicates a skew in the data, potentially explaining the lack of predictive utility for this variable. Whilst the histology dummy variable was still skewed (62 epithelioid to 24 non-epithelioid) it was less so than the other variables explained previously. An extended analysis (**Supplementary File 3**) divided patients into “more malignant” and “less malignant” using two independent analyses as a proxy: firstly, division by lymph node involvement and secondly (separately) division by metastatic status. No differential abundance in microbiome signatures between the lymph node groups was observed (based on q-value), and only one genus was differentially abundant based on metastatic state (*Bromovirus*); however, this genus was not present on the list of 107 genera linked to survival (**Supplementary File 3**). As such, it may be that the genera influence survival through mechanisms outside of malignant state (/lymph node involvement/metastasis).

Further to the above, the identification that epithelioid histology was a significant prognostic indicator compared to other variables has evidence in the literature (46). As Petersen and colleagues published in 2021, epithelioid histology was the only positive independent prognostic factor for treated pleural mesothelioma patients (46). In this patient cohort, neither age nor gender nor stage were significant by univariate analysis for overall survival (OS). It should be noted that another group in the same study (46), those receiving best supportive care (BSC) rather than anti-tumor treatment, did demonstrate, *via* univariate analysis, significant association for gender (female), epithelioid histology, and performance status. However, stage was significant for the BSC group only at the p<0.1 level, not p<0.05 level, thus indicating general agreement between the results by Petersen (46) and the results presented in this article. It is evident that the potential prognostic value of the microbiome should be explored further.

It is also recognized that tumor-associated macrophages have an impact on mesothelioma prognosis, with the presence of M2-like macrophages leading to worse outcomes (47). As such, given the importance of the microbiome identified herein, it would be interesting to investigate any potential links between M2 macrophages, the microbiome, and mesothelioma. However, as of 17^th^ February 2023, there were zero articles returned on PubMed for a basic Boolean search of this (search terms: (Mesothelioma) AND ((M2-like macrophages) OR (M2 macrophages)) AND (microbiome)). Looking into the wider literature also yielded limited results; only sixteen articles were returned for a search for these terms without mesothelioma on the 17^th^ February 2023 (search terms: (Microbiome[Title/Abstract]) AND ((M2-like macrophages[Title/Abstract]) OR (M2 macrophages[Title/Abstract]))), dropping to nine when “cancer” was added as a search term (without the Title/Abstract] filter). That said, despite the limited literature, some valuable insights are present. Examples of the microbiome affecting M2 macrophages include positive effects of *Lactobacillus murinus* on the reduction of intestinal injury in mice *via* stimulation of IL-10 release from macrophages (48) and stimulation of tissue remodelling through M2 macrophages in inflammatory bowel disease by *Clostridium innocuum* (a gut bacteria) (49). *Clostridium butyricum*-derived extracellular vesicles affect repolarization of M2 macrophages and protect against colitis (50). In extramammary Paget’s disease high levels of *Staphylococcus aureus* were detected that coincided with CD163-positive M2-like macrophages (51), whereas potential association of *Shewanella*, *V. parahaemolyticus*, and *Microbacterium* sp. with prostate cancer has been described, with indications that malignant tissue has higher proportion of M2 microphages (52). High risk colon cancer patients were shown to have increased proportion of M2 macrophages (53), whereas *Fusobacterium nucleatum* is negatively associated with M2 macrophages and positively associated with better outcome in patients with oral squamous cell carcinoma (54). As highlighted, there were no mesothelioma-specific articles returned on this topic, and half of the articles found from the wider search were published in 2020 or later, again indicating this field’s relative infancy. The importance of immune infiltration and inflammation lead to a supplementary analysis involving GeneCards (55, 56), where the differentially expressed genes between the Low and High Survivors were compared to the top 5% of genes involved in each process (**Supplementary File 4**). However, there was minimal overlap between the genes involved in each process and the differentially expressed genes (3/334), indicating that further exploration is required.

Complementing the microbiome analysis was the differential gene expression and functional annotation analyses. Through this, 60 genes were identified to be upregulated in high surviving patients, whilst 274 were upregulated in low surviving patients. The functional annotation analysis also generated insight, with the low surviving group having enriched annotations in terms relating to the cell cycle, cell division, and DNA repair. These processes, if upregulated and deregulated, could potentially explain the poor survival rate of these patients. Comparatively, the most enriched term (according to Metascape) for the high surviving patients was fatty acid metabolic process. Of note is that the high surviving patients had 103 genera signatures more abundant than in the low surviving patients, versus four genera signatures in the reverse direction. This could be interpreted as the high survivors having more abundant microbiome in general, or at least a higher proportion of certain genera in their microbiome composition. Building on this, there are published links between dietary lipids/lipid metabolism and the gut microbiota (57). Fatty acids have the ability to lyse and solubilize bacterial cell membranes (57–59) whilst the gut microbiome may influence lipid metabolism. The links between lipids and gut microbiota have been comprehensively reviewed (57) and although the present study examined the cancer intratumor microbiome rather than the gut microbiome, the fact that “fatty acid metabolic process” was the most enriched term in the group which most genera were increased in demonstrates a potentially direct link between the microbiota/microbiome signatures, the differentially expressed genes and annotations, and the patient survival. Furthermore, it is intriguing that *Klebsiella*, whose signature was more abundant in “Low Survivors”, is known to modify its lipopolysaccharide to evade immune surveillance, in the lungs of mice (60). Thus, this demonstrates further potential linkage between the microbiome and cancer, as evading immune detection is a known cancer hallmark (61). Although the association between these genera and fatty acid metabolism in MMe could be correlational rather than causative, we believe it certainly lays the groundwork for further studies to investigate these in more detail.

Although age was not found to be an independent predictor of survival, we observed that the low survivor group tended to have a higher age at diagnosis (median age 66 versus 62, with standard deviations of 11.52 and 7.86 respectively). This, although not statistically significant, may be due to a generally worse clinical status of older patients, but it is interesting to note that ageing affects the microbiota composition and, in turn, the microbiome impacts on organismal ageing and lifespan (62). Indeed, microbiome dysbiosis has been proposed as an additional hallmark and biomarker of aging (62). Ageing is associated with a reduced microbiome diversity and with commensals which favor inflammageing and impair immune functions (63, 64). Compared with the healthy elderly, frail elderly people host more proinflammatory Bacteroidetes commensals and fewer producers of beneficial short-chain fatty acids (65), which is notable given the high surviving group in this study, who could be argued to have ‘more’ intratumor microbiota due to abundance differences, had fatty acid metabolism as the most enriched biological function. A recent study performed on the duodenal microbiome of elderly patients showed that beyond chronological age, also the number of concomitant diseases and the number of medications affected the microbiome composition with the latter increasing the presence of *Klebsiella* (65). Taken together, such evidence seems consistent with the scenario that we unveiled analyzing the tumor microbiome in mesothelioma patients, which deserves further investigation.

A key limitation of this article is that only pleural mesothelioma has been explored. Indeed, it is recognized that the different subtypes of mesothelioma—pleural, pericardial, peritoneal and testicular—may have different underlying development mechanisms and response to stimuli e.g. a difference in the response to asbestos was noted between peritoneal and pleural mesothelioma (66). Regrettably, cBioPortal (19, 20) had no available information on peritoneal mesothelioma patients. As such, a key area for further exploration would be the investigation in this mesothelioma subtype.

In summary, this article has identified 107 cancer microbiome genera that are pertinent to MMe patient survival, which opens avenues for a new research area in this under-researched cancer. Furthermore, the microbiome was validated in this article as being important for survival through two separate approaches (multiple linear regression modelling and Cox proportional hazards modelling), both of which recognized it as more statistically significant than patient age, tumor stage and even histology (though the effect size of histology remained greater due to its hazard ratio). Laboratory analyses, for example *in vitro* co-culture methods, could be used to start generating solid mechanistic insight at the preclinical level. This foundation will improve understanding of how the microbiome is relevant in MMe and may lead to improved patient outcomes.

## Data availability statement

The original contributions presented in the study are included in the article/**Supplementary Material**. Further inquiries can be directed to the corresponding authors.

## Author contributions

Conceptualization: EB; Data curation: FP, MK-D, CC, LM, and EB; Funding Acquisition: LM; Investigation: FP, MK-D, CC, LM, and EB; Project Administration: LM and EB; Validation: FP, MK-D, CC, LM, and EB; Writing: FP, MK-D, CC, LM, and EB. All authors contributed to the article and approved the submitted version.

## Supplementary material

Supplementary Figure 1 Comparison of age between “High Survivors” and “Low Survivors”

Supplementary Figure 2 Comparison of metastatic state (TNM value) between “High Survivors” and “Low Survivors”

Supplementary Figure 3 Comparison of tumor size (TNM value) between “High Survivors” and “Low Survivors”

Supplementary Figure 4 Comparison of lymph node involvement (TNM value) between “High Survivors” and “Low Survivors”

Supplementary Figure 5 Comparison of cancer stage (number staging system) between “High Survivors” and “Low Survivors”

Supplementary Figure 6 Comparison of biological sex between “High Survivors” and “Low Survivors”

Supplementary Figure 7 Comparison of histological subtype between “High Survivors” and “Low Survivors”. Note that per the World Health Organization (https://apps.who.int/iris/bitstream/handle/10665/96612/9789241548496_eng.pdf), 9050/3 refers to “Mesothelioma, malignant, NOS”, 9052/3 to “Epithelioid mesothelioma, malignant, NOS”, and 9053/3 to “Mesothelioma, biphasic, malignant, NOS”.

Supplementary File 1 Excel file of DEGs and DAVID analysis.

Supplementary File 2 Excel file of multiple linear regression inputs and iterations, as well as clinical data for the patients.

Supplementary File 3 Excel file of microbiome abundance analysis based on patient subdivision by malignancy

Supplementary File 4 Excel file of differentially expressed genes mapped to genes involved in either immune infiltration and inflammation (per GeneCards (55, 56)).
