# Correction: Intratumor microbiota as a novel potential prognostic indicator in mesothelioma

**DOI:** 10.3389/fimmu.2025.1703111

**Published:** 2025-10-15

**Authors:** Francesca Pentimalli, Marija Krstic-Demonacos, Caterina Costa, Luciano Mutti, Emyr Yosef Bakker

**Affiliations:** ^1^ Department of Medicine and Surgery, LUM University “Giuseppe DeGennaro”, Bari, Italy; ^2^ Biomedical Research Centre, School of Science, Engineering and Environment, University of Salford, Salford, United Kingdom; ^3^ Cell Biology and Biotherapy Unit, Istituto Nazionale Tumori-Scientific Institute for Research and Care (IRCCS)-Fondazione G. Pascale, Napoli, Italy; ^4^ Center for Biotechnology, Sbarro Institute for Cancer Research and Molecular Medicine, College of Science and Technology, Temple University, Philadelphia, PA, United States; ^5^ Department of Biotechnological and Applied Clinical Sciences, University of Aquila, L’Aquila, Italy; ^6^ School of Medicine, University of Central Lancashire, Preston, United Kingdom

**Keywords:** mesothelioma, microbiota, microbiome, bioinformatics, Kaplan-Meier, DEG (differentially expressed gene) analysis, functional annotation analysis, Cox proportional hazards modelling

An underpinning article cited in the original version of this manuscript [Poore et al, 2020 (https://www.nature.com/articles/s41586-020-2095-1)] was retracted in June 2024. This was on the basis of criticisms published by Gihawi and colleagues in 2023 (https://pubmed.ncbi.nlm.nih.gov/37811944/), which occurred after the publication of the original version of this manuscript. Whilst the rebuttal by Sepich-Poore and colleagues remains live to this date (https://www.nature.com/articles/s41388-024-02974-w), the retraction of the 2020 article may impact the validity of some specific information in our article (e.g. some exact genera), though the original overarching conclusions may still hold merit.

A correction has been made to the section **Methods**, *Identifying microbiome differences*, Paragraph 2:

“This analysis automatically generated the Kaplan-Meier survival curve between the two groups, alongside the microbiome signatures comparison. In order to calculate a more precise p-value alongside the hazard ratio for the survival data, the resultant raw Kaplan-Meier data was downloaded and input to KMPlot using the upload function (23, 24). The microbiome signatures data were originally added to cBioPortal for a number of cancers on the basis of another study, which has since been retracted based on criticisms from Gihawi and colleagues (https://pubmed.ncbi.nlm.nih.gov/37811944/). Although the authors’ rebuttal in which they said they reproduced their findings remains live (https://www.nature.com/articles/s41388-024-02974-w), it is worth noting that this retraction means that some specific information in this manuscript (e.g. exact genera) may be uncertain, even if there may still be merit in the overarching findings.

Clinical parameters for patients were also obtained via the Compare analysis, as were the differentially expressed genes.”

A correction has been made to the section **Methods**, *Contaminant removal*, Paragraphs 1 and 2:

“Due to the recognised issue of contaminants (i.e., tumour sample contamination by external microbes during data collection and processing) when considering microbiome data, a decontamination analysis was performed on the list of genera that were statistically significantly associated (based on q-value) with patient survival.

This was performed through cross-examination with supplementary information from the aforementioned original article which added the signatures to cBioPortal.”

The original version of this article has been updated.

