# Supplementary figures and images for: Intratumor microbiota as a novel potential prognostic indicator in mesothelioma
Source: Front Immunol. 2023 Mar 14;14:1129513. doi: 10.3389/fimmu.2023.1129513 (PMC10043377; doi:10.3389/fimmu.2023.1129513)

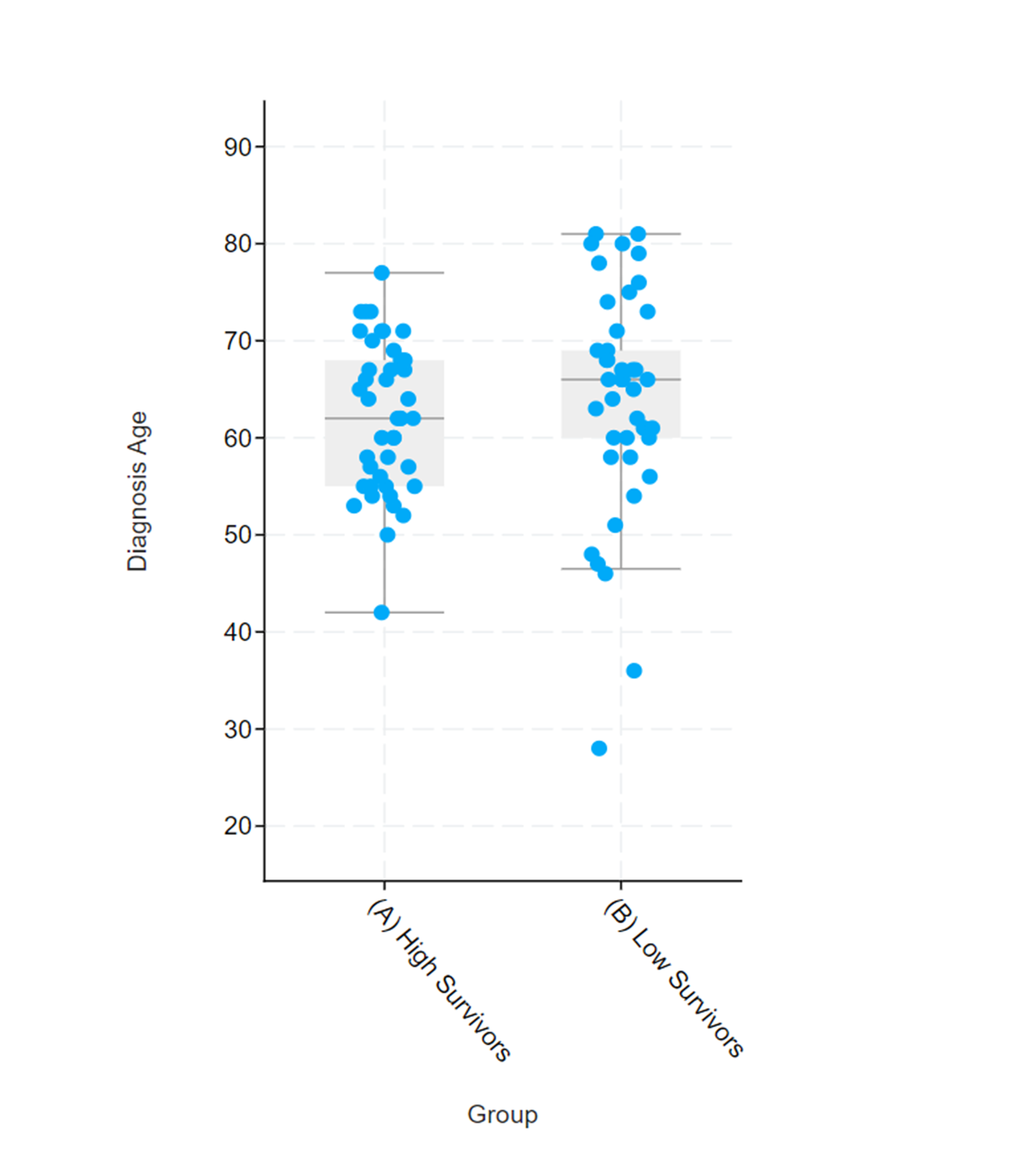

Supplement: Supplementary Figure 1 — Comparison of age between “High Survivors” and “Low Survivors” [file Image_1.tif]

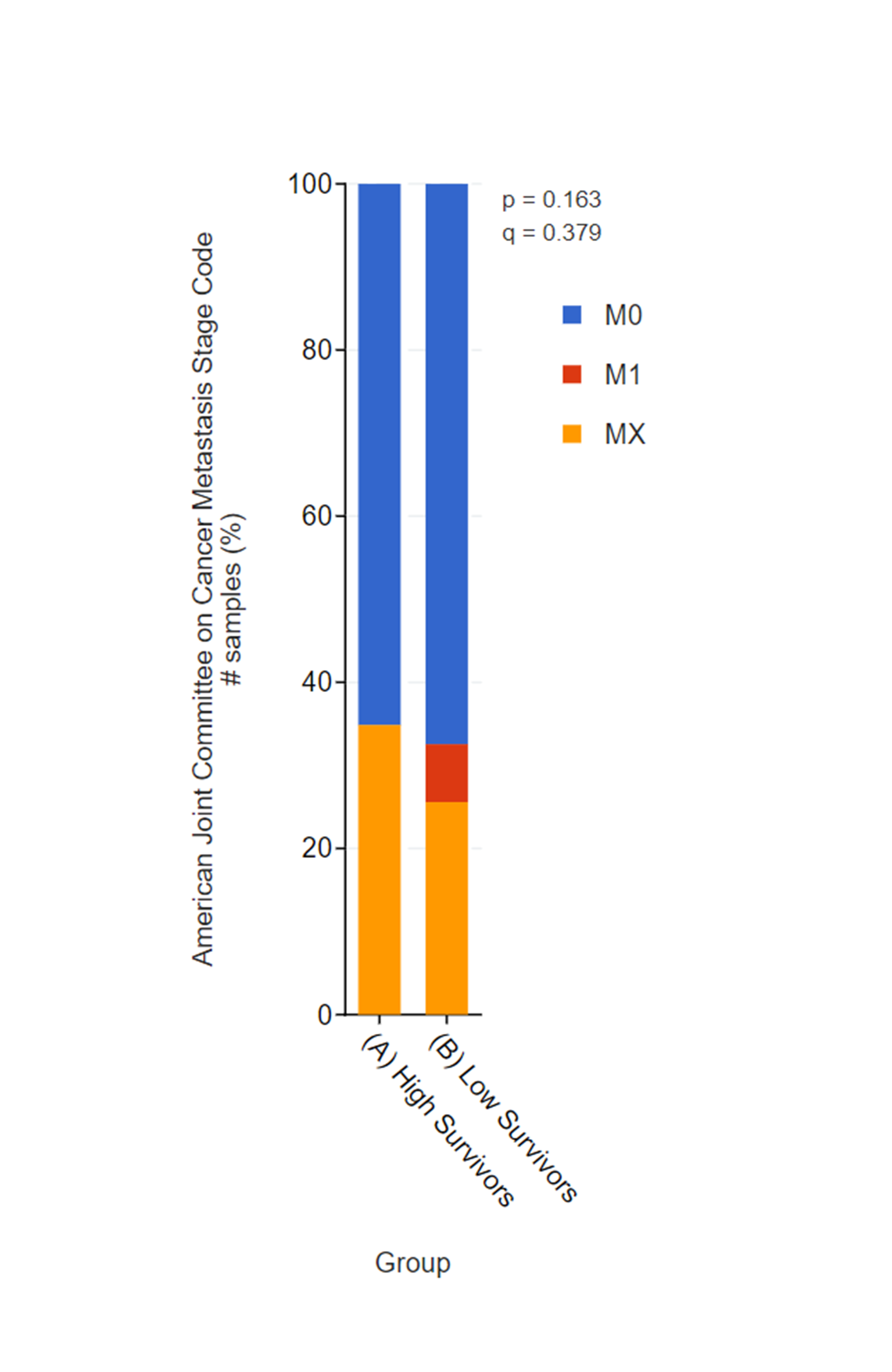

Supplement: Supplementary Figure 2 — Comparison of metastatic state (TNM value) between “High Survivors” and “Low Survivors” [file Image_2.tif]

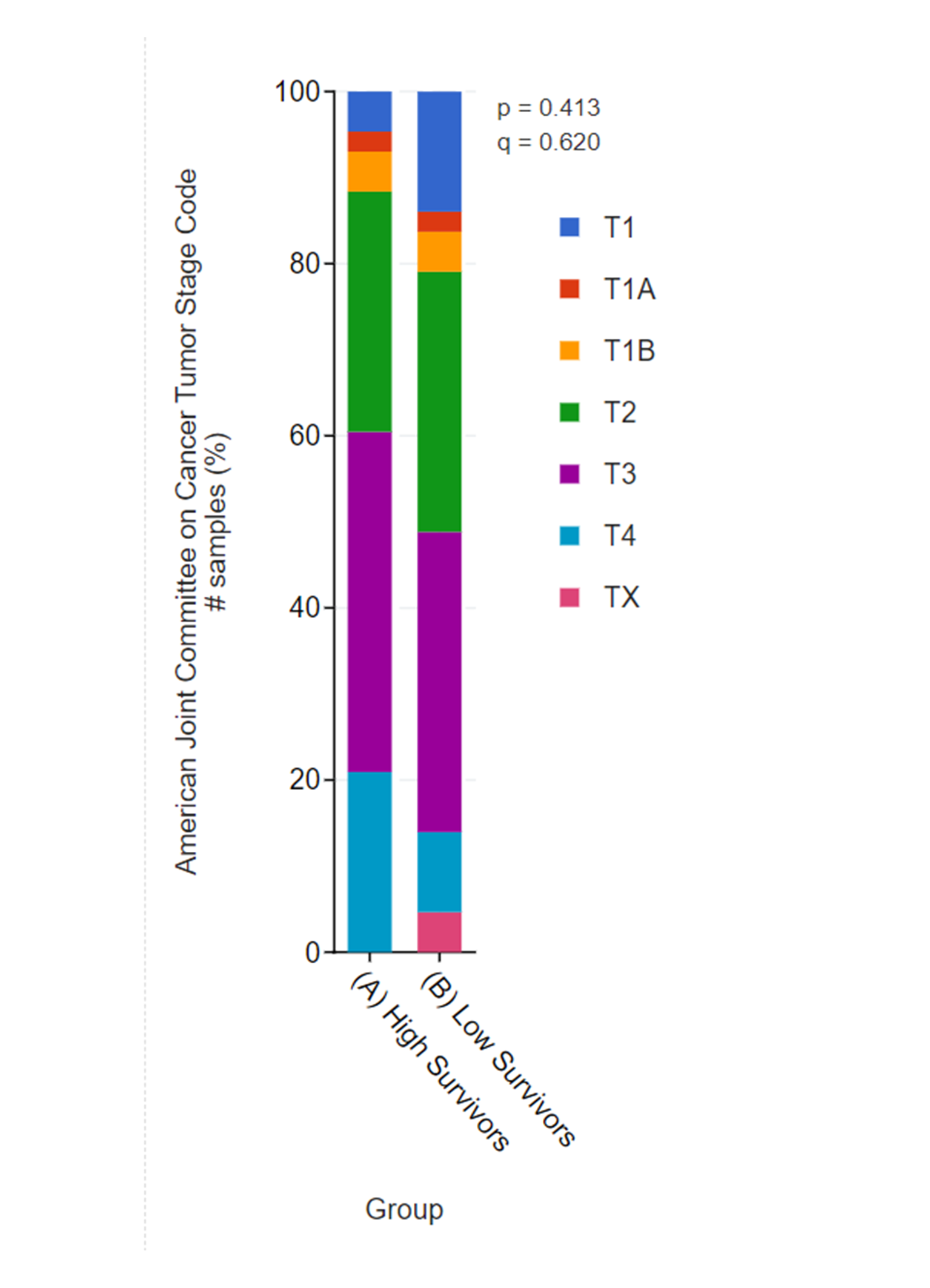

Supplement: Supplementary Figure 3 — Comparison of tumor size (TNM value) between “High Survivors” and “Low Survivors” [file Image_3.tif]

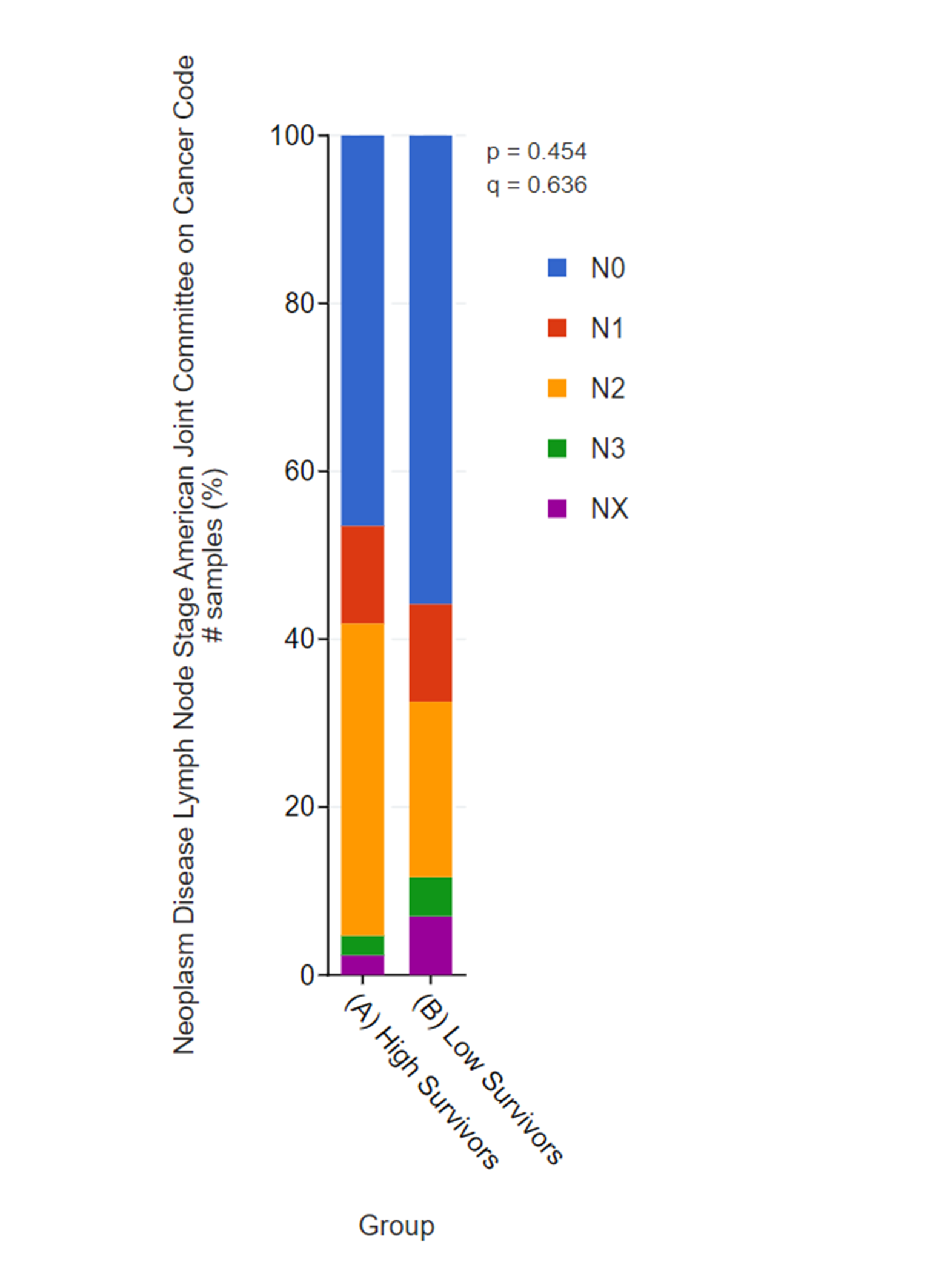

Supplement: Supplementary Figure 4 — Comparison of lymph node involvement (TNM value) between “High Survivors” and “Low Survivors” [file Image_4.tif]

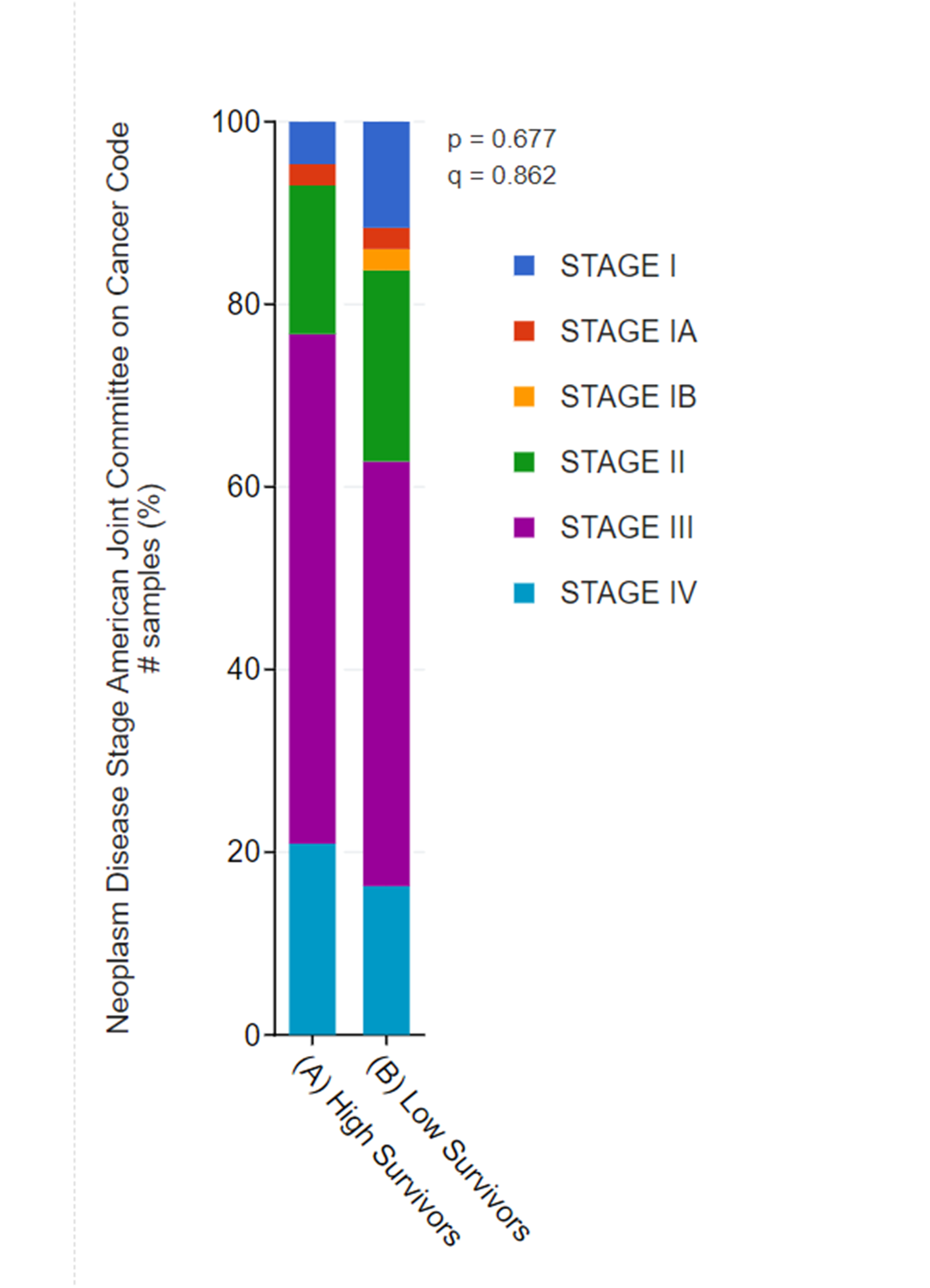

Supplement: Supplementary Figure 5 — Comparison of cancer stage (number staging system) between “High Survivors” and “Low Survivors” [file Image_5.tif]

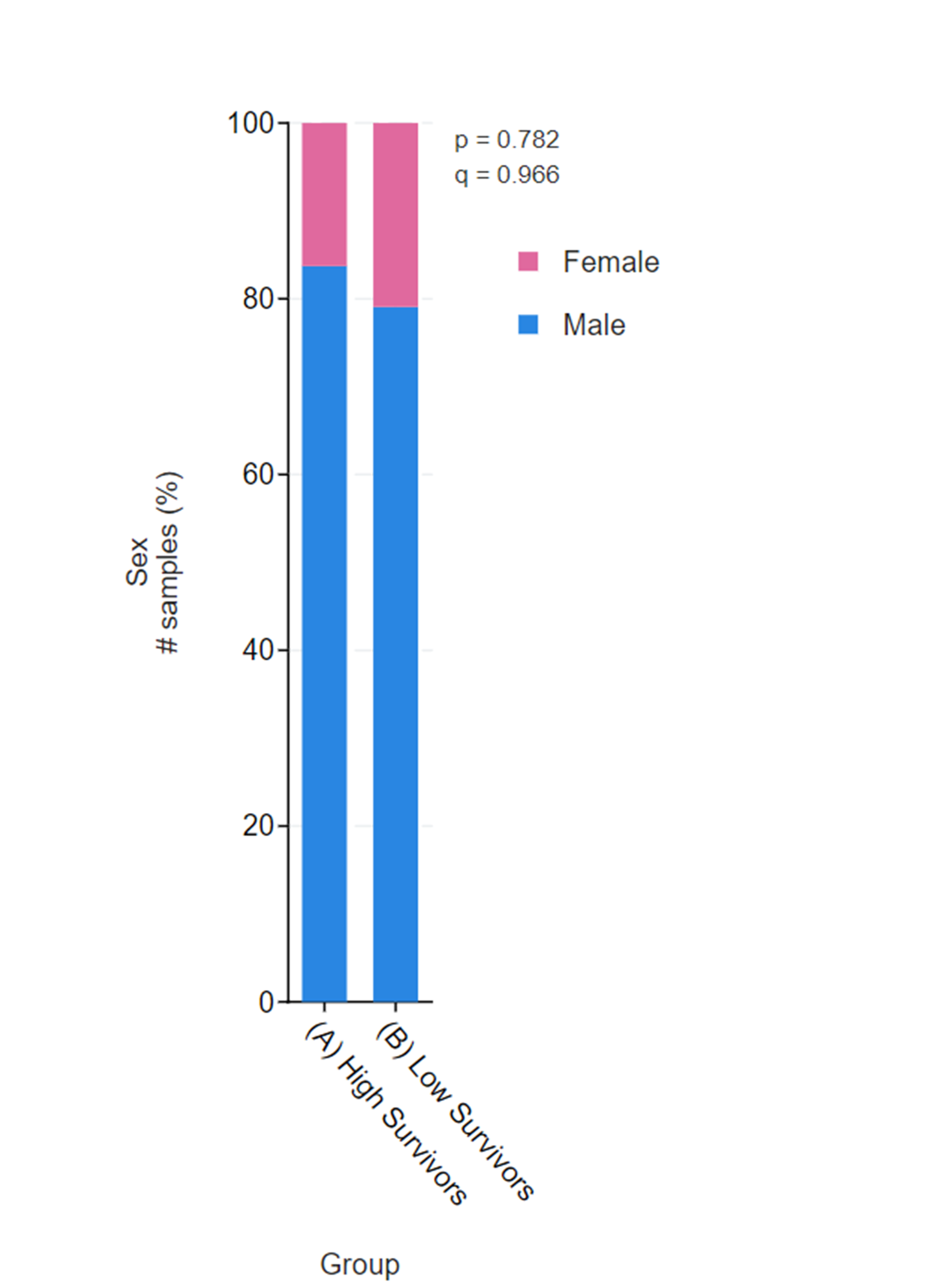

Supplement: Supplementary Figure 6 — Comparison of biological sex between “High Survivors” and “Low Survivors” [file Image_6.tif]

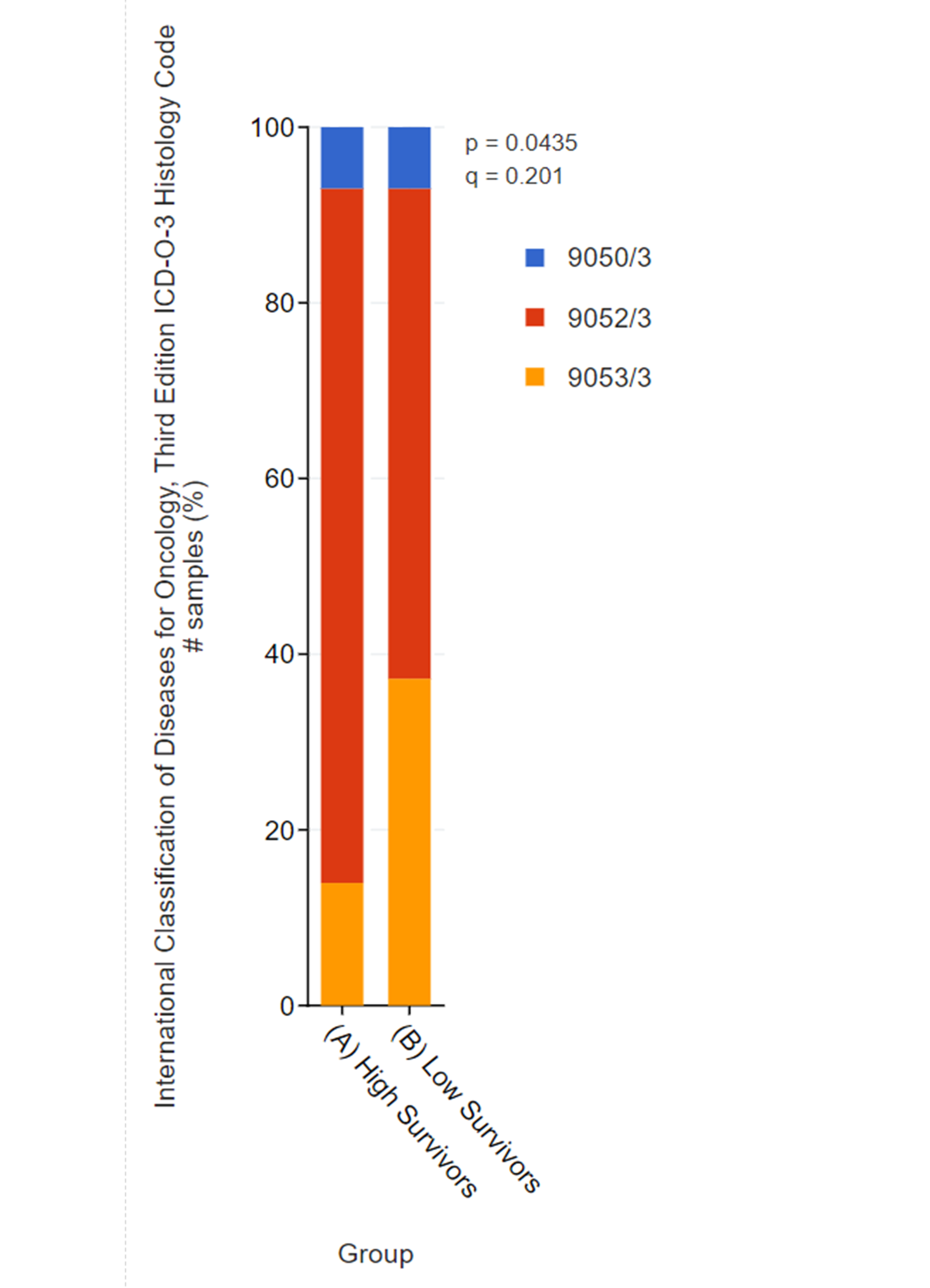

Supplement: Supplementary Figure 7 — Comparison of histological subtype between “High Survivors” and “Low Survivors”. Note that per the World Health Organization (https://apps.who.int/iris/bitstream/handle/10665/96612/9789241548496_eng.pdf), 9050/3 refers to “Mesothelioma, malignant, NOS”, 9052/3 to “Epithelioid mesothelioma, malignant, NOS”, and 9053/3 to “Mesothelioma, biphasic, malignant, NOS”. [file Image_7.tif]
