# Supplementary material for: Intratumor microbiota as a novel potential prognostic indicator in mesothelioma
Source: Front Immunol. 2023 Mar 14;14:1129513. doi: 10.3389/fimmu.2023.1129513 (PMC10043377; doi:10.3389/fimmu.2023.1129513)
Supplement: Supplementary file 12 [file Table_1.docx]

**Supplementary Table 1**

*Supplementary Table 1: Microbiome literature assessment. Of all the genera, only* Klebsiella *returned a result for it and mesothelioma, as highlighted by the asterisk (*).*

| **Entity Name** | **Higher in** | **PubMed Entries for Genus & Mesothelioma** | **PubMed Entries for Genus & Cancer** |
| --- | --- | --- | --- |
| **Ponticaulis** | High Survivors | 0 | 0 |
| **Kosmotoga** | High Survivors | 0 | 0 |
| **Vulgatibacter** | High Survivors | 0 | 0 |
| **Youngiibacter** | High Survivors | 0 | 0 |
| **Candidatus_Ishikawaella** | High Survivors | 0 | 0 |
| **Magnetococcus** | High Survivors | 0 | 4 |
| **Stanieria** | High Survivors | 0 | 0 |
| **Euryhalocaulis** | High Survivors | 0 | 0 |
| **Marinimicrobium** | High Survivors | 0 | 0 |
| **Pseudohongiella** | High Survivors | 0 | 0 |
| **Leucothrix** | High Survivors | 0 | 0 |
| **Microchaete** | High Survivors | 0 | 2 |
| **Cetobacterium** | High Survivors | 0 | 2 |
| **Thermodesulfobium** | High Survivors | 0 | 0 |
| **Actinopolymorpha** | High Survivors | 0 | 1 |
| **Marinagarivorans** | High Survivors | 0 | 0 |
| **Hydrogenivirga** | High Survivors | 0 | 0 |
| **Candidatus_Evansia** | High Survivors | 0 | 0 |
| **Criblamydia** | High Survivors | 0 | 0 |
| **Cycloclasticus** | High Survivors | 0 | 1 |
| **Leptonema** | High Survivors | 0 | 4 |
| **Robiginitalea** | High Survivors | 0 | 0 |
| **Paraoerskovia** | High Survivors | 0 | 0 |
| **Methylomarinum** | High Survivors | 0 | 0 |
| **Allofustis** | High Survivors | 0 | 0 |
| **Salisaeta** | High Survivors | 0 | 0 |
| **Thiocapsa** | High Survivors | 0 | 0 |
| **Methylohalobius** | High Survivors | 0 | 0 |
| **Mesonia** | High Survivors | 0 | 0 |
| **Pragia** | High Survivors | 0 | 4 |
| **Agarivorans** | High Survivors | 0 | 0 |
| **Porticoccus** | High Survivors | 0 | 0 |
| **Haematospirillum** | High Survivors | 0 | 0 |
| **Beggiatoa** | High Survivors | 0 | 1 |
| **Neptunomonas** | High Survivors | 0 | 0 |
| **Halorhodospira** | High Survivors | 0 | 0 |
| **Desulfospira** | High Survivors | 0 | 0 |
| **Puniceibacterium** | High Survivors | 0 | 0 |
| **Simiduia** | High Survivors | 0 | 0 |
| **Marivirga** | High Survivors | 0 | 0 |
| **Salinibacter** | High Survivors | 0 | 0 |
| **Salegentibacter** | High Survivors | 0 | 0 |
| **Ammonifex** | High Survivors | 0 | 0 |
| **Nesiotobacter** | High Survivors | 0 | 0 |
| **Gulosibacter** | High Survivors | 0 | 0 |
| **Thalassospira** | High Survivors | 0 | 1 |
| **Pleurocapsa** | High Survivors | 0 | 1 |
| **Anaerofustis** | High Survivors | 0 | 1 |
| **Yatapoxvirus** | High Survivors | 0 | 49 |
| **Leeia** | High Survivors | 0 | 10 |
| **Hylemonella** | High Survivors | 0 | 0 |
| **Nautilia** | High Survivors | 0 | 0 |
| **Thiolapillus** | High Survivors | 0 | 0 |
| **Azovibrio** | High Survivors | 0 | 0 |
| **Closterovirus** | High Survivors | 0 | 3 |
| **Sharpea** | High Survivors | 0 | 0 |
| **Halobacteroides** | High Survivors | 0 | 0 |
| **Oleiphilus** | High Survivors | 0 | 0 |
| **Chelonobacter** | High Survivors | 0 | 0 |
| **Candidatus_Methylopumilus** | High Survivors | 0 | 0 |
| **Dichelobacter** | High Survivors | 0 | 1 |
| **Phikzlikevirus** | High Survivors | 0 | 0 |
| **Cyclobacterium** | High Survivors | 0 | 64 |
| **Desulfonatronospira** | High Survivors | 0 | 0 |
| **Yokenella** | High Survivors | 0 | 1 |
| **Candidatus_Photodesmus** | High Survivors | 0 | 0 |
| **Candidatus_Arthromitus** | High Survivors | 0 | 4 |
| **Hydrogenobacter** | High Survivors | 0 | 0 |
| **Sphingorhabdus** | High Survivors | 0 | 0 |
| **Ventosimonas** | High Survivors | 0 | 0 |
| **Dinoroseobacter** | High Survivors | 0 | 0 |
| **Paludibacterium** | High Survivors | 0 | 0 |
| **Salinispira** | High Survivors | 0 | 0 |
| **Crinivirus** | High Survivors | 0 | 1 |
| **Thioalkalimicrobium** | High Survivors | 0 | 1 |
| **Acidihalobacter** | High Survivors | 0 | 0 |
| **Chelativorans** | High Survivors | 0 | 2 |
| **Methylosarcina** | High Survivors | 0 | 0 |
| **Amycolicicoccus** | High Survivors | 0 | 0 |
| **Sulfuricurvum** | High Survivors | 0 | 2 |
| **Acaricomes** | High Survivors | 0 | 0 |
| **Gemmata** | Low Survivors | 0 | 1 |
| **Halolactibacillus** | High Survivors | 0 | 0 |
| **Desulfobacula** | High Survivors | 0 | 0 |
| **Cafeteriavirus** | High Survivors | 0 | 0 |
| **Thioflavicoccus** | High Survivors | 0 | 0 |
| **Nitratifractor** | High Survivors | 0 | 0 |
| **Betaentomopoxvirus** | High Survivors | 0 | 0 |
| **Klebsiella*** | Low Survivors | 3 | 1609 |
| **Vagococcus** | High Survivors | 0 | 3 |
| **Lambdalikevirus** | High Survivors | 0 | 387 |
| **Halotalea** | High Survivors | 0 | 0 |
| **Sanguibacter** | Low Survivors | 0 | 0 |
| **Limnochorda** | High Survivors | 0 | 0 |
| **Desulfatirhabdium** | High Survivors | 0 | 0 |
| **Aliiroseovarius** | High Survivors | 0 | 0 |
| **Achromobacter** | Low Survivors | 0 | 60 |
| **Oscillochloris** | High Survivors | 0 | 0 |
| **Sneathiella** | High Survivors | 0 | 0 |
| **Heliobacterium** | High Survivors | 0 | 0 |
| **Jejuia** | High Survivors | 0 | 0 |
| **Aliiglaciecola** | High Survivors | 0 | 0 |
| **Pelodictyon** | High Survivors | 0 | 0 |
| **Desulfobacca** | High Survivors | 0 | 0 |
| **Gracilibacillus** | High Survivors | 0 | 0 |
| **Bermanella** | High Survivors | 0 | 0 |
| **Methylacidiphilum** | High Survivors | 0 | 0 |
